# The Contribution of Electron Paramagnetic Resonance to Melanoma Research

**DOI:** 10.1155/2011/273280

**Published:** 2011-09-20

**Authors:** Quentin Godechal, Bernard Gallez

**Affiliations:** Biomedical Magnetic Resonance Research Group, Louvain Drug Research Institute (LDRI/REMA), Université Catholique de Louvain, Avenue Mounier 73.40, 1200 Brussels, Belgium

## Abstract

The incidence of malignant melanoma, the most dangerous form of skin cancer, is rising each year. However, some aspects of the tumor initiation and development are still unclear, and the current method of diagnosis, based on the visual aspect of the tumor, shows limitations. For these reasons, developments of new techniques are ongoing to improve basic knowledge on the disease and diagnosis of tumors in individual patients. This paper shows how electron paramagnetic resonance (EPR), a method able to detect free radicals trapped in melanin pigments, has recently brought its unique value to this specific field. The general principles of the method and the convenience of melanin as an endogenous substrate for EPR measurements are explained. Then, the way by which EPR has recently helped to assess the contribution of ultraviolet rays (UVA and UVB) to the initiation of melanoma is described. Finally, we describe the improvements of EPR spectrometry and imaging in the detection and mapping of melanin pigments inside *ex vivo* and *in vivo* melanomas. We discuss how these advances might improve the diagnosis of this skin cancer and point out the present capabilities and limitations of the method.

## 1. Introduction

Malignant melanoma is a skin tumor characterized by the uncontrolled proliferation of melanocytes. This tumor is the most dangerous form of skin cancer and is responsible of 3 of 4 deaths linked to a skin cancer. The incidence of malignant melanoma is rising each year [1, 2], and, nowadays, the cumulative lifetime risk for an invasive melanoma is estimated at 1/59 in the USA A link between sunlight exposure and melanoma causation has been for a long time established [3–5]; however, it is still not clear how the different ultraviolet wavelengths (UVA and UVB) are implicated [6]. The role of melanin as a possible endogenous photosensitizer is also subject to discussion. This discussion and the way that a new measurement method based on electron paramagnetic resonance (EPR) might answer to it will be explained and debated.

The abilities and perspectives of EPR spectrometry and imaging as a detection method for melanin pigments, and consequently for pigmented melanomas, will be also discussed. Indeed, for now the only method used by dermatologists for suspecting melanoma is an optical examination of the lesion based on the detection of five factors: Asymmetry, irregular Borders, nonhomogenous Color, large Diameter, and Evolving (also known as the ABCDE rule). This optical examination, even if effective, shows limitations: it does not provide any information about the penetration of the tumor in the skin, which is a crucial factor to determine the growth state of the potential melanoma and to provide an adapted treatment. The use of EPR imaging for the mapping of free radicals trapped in melanin might allow filling in this lack of spatial information in a noninvasive way.

## 2. A Few Words about Melanin(s)

Melanin is the most widespread pigment in the epithelia of vertebrates. This molecule is responsible of the pigmentation of skin, hair, and eye. Historically, its name comes from the Greek word *melanos* that means “dark”. The origin of this name is generally attributed to the Swedish chemist Berzelius [7]. As explained by Riley in his review about melanin [8], the term “melanin” has been used fairly indiscriminately to mean any dark pigment but the nomenclature has been refined in the case of mammalian melanin. Indeed, later, two major different kinds of melanin have been described: eumelanin and pheomelanin. Eumelanin is a brown-black pigment derived from the enzymatic oxidation of tyrosine through 3,4-dihydroxyphenylalanine (dopa) [9], while pheomelanin is a yellow reddish-brown pigment following a similar synthesis pathway but including cysteine [10]. However, it is generally accepted that pure eumelanin or pure pheomelanin is rare in normal tissues. Most of the time, eumelanin and pheomelanin monomers are linked in various proportions to create the melanin macromolecules [11, 12]. 

**Figure 1:**
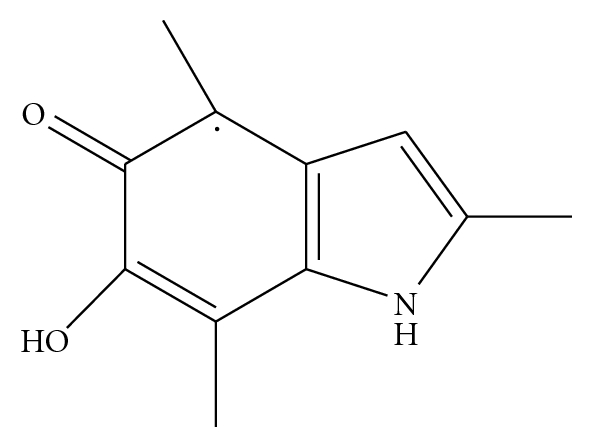
Semiquinone radical trapped in melanin, responsible of the paramagnetic properties of the molecule.

The melanin polymer has some interesting properties among which light absorbance, redox properties, and chelating properties [8]. The wide spectral absorbance of light is linked to the high degree of conjugation in the molecule. The redox properties, especially of eumelanins, are caused by the delocalization of an electron between orthoquinone and catecholic moieties, which give rise to semiquinone free radicals (Figure 1). Due to these radicals, melanins can take part in one-electron and two-electron redox reactions. This property is responsible of a photooxidation reaction when the pigment absorbs light and of the photosensitizer role of melanin, role that will be explained in a dedicated paragraph of this paper. Moreover, the semiquinone free radicals trapped in melanin are detectable using EPR. As we will see later, this makes EPR a unique tool to assess the extension of melanoma.

## 3. Paramagnetism and Electron Paramagnetic Resonance (EPR)

Electron paramagnetic resonance (EPR) is a magnetic resonance method similar to nuclear magnetic resonance (NMR) which focuses on the detection of paramagnetic materials. The paramagnetism notion refers to the behavior of a material substrate that does not possess spontaneous magnetization but, under an external magnetic field, acquires a magnetization parallel to this magnetic field. This phenomenon, explained by the quantum physics principles [13], results from the properties of the electron spin [14]. It is important to note that only the unpaired electrons might move from a low energy level to a higher (and vice versa) and enter into resonance. EPR spectrometry is a method that detects the absorption of energy linked to the resonance phenomenon. The quantity of this energy differs in function of the kind of radicals and their environment. Consequently, this technique is able to detect and characterize free radicals by providing spectra which are specific to the radicals detected.

Although the EPR technique is similar to NMR, there are two notable differences between them [16]. First, the gyromagnetic ratio of an unpaired electron is largely higher than that of a proton. This means that the standard EPR spectrometers have to operate at much higher frequencies and lower fields than conventional NMR spectrometers. When working at such high frequencies (typically, 9 GHz, which is a standard frequency used in the most usual EPR spectrometer), the nonresonant absorption of the electromagnetic radiation by the liquid water of the biological samples presents a serious problem. At 9 GHz (X-Band mode), the microwaves will only penetrate up to 1 mm inside a biological water-containing sample. Larger aqueous samples or animals can be studied only by reducing the operating frequency to 1 GHz (L-Band mode) or less. At this frequency, the penetration of microwaves is about 1 cm, which can be convenient for studies on small samples, small animals, or superficial tissues such as skin. However, this results in an important loss of sensitivity.

The second major difference between EPR and NMR comes from the difference of their relaxation time, which is in the range of nanoseconds for electron and in the range of milliseconds for proton. This has two consequences: first, *in vivo* EPR spectra are mostly obtained through continuous wave (CW) experiments, whereas, in clinical MRI, results are always obtained with the use of pulsed radiowave. Second, compared to MRI, EPR imaging requires a gradient field at least one order of magnitude greater.

During the last 60 years, EPR spectrometry has been successfully used in many parts of the scientific research, especially in biology where it contributed to the understanding of a large number of physiological processes. The fields where EPR has been the most used and useful already possess their own review(s), such as the study of iron-containing molecules [17], the assessment of oxygen concentration in tumors [16, 18], the detection of reactive nitrogen and oxygen species *in vivo* [19, 20], or the assessment of redox state in biological tissues *in vivo *[21].

A more recent method derived from EPR spectroscopy, called EPR imaging, consists in adding an external gradient field that modulates the resonance frequency of the paramagnetic species in function of their position in space [22, 23]. As a consequence, it is possible to get 2D and 3D maps of the distribution of free radicals. This ability has, for example, been used, alone or coupled, with another technology, to improve our knowledge of the brain redox state [24], measure venous and arterial oxygenation [25], monitor the evolution of tumor oxygenation after treatment [26, 27], or map the repartition of radio-induced free radicals after irradiation [28].

**Figure 2:**
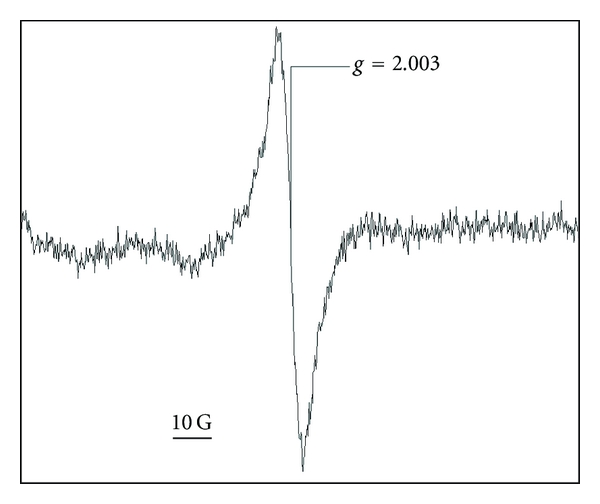
Typical EPR spectrum of melanin. This spectrum was obtained for measurement of 5 *μ*g of synthetic melanin.

Because in 1954, melanin was found to contain stable free radicals detectable by EPR [29] and that these radicals provided a specific EPR spectrum (Figure 2), this molecule became a very interesting substrate for further spectrometry and imaging studies. For a long time, these two methods, and especially spectrometry, have been used to try to determine the structure of melanin, to understand the mechanisms behind its protective role against sunlight aggressions, and to improve our knowledge of melanoma.

## 4. Melanin, UV Rays, and EPR

An excellent extensive review of this topic (putting together melanin pigments, UV irradiation, and EPR spectrometry) has been published by Lund and Timmins in 2007 [6]. We briefly summarize here the research advances in this field. The role of melanin in the protection of skin from light radiations has been generally accepted for a long time. Indeed, a link between melanoma causation and exposure to sunlight has been observed [3–5]. However, it is unclear how ultraviolet (UV) rays of sunlight might cause melanoma. The two major types of UVs, UVA and UVB, seem to have different effects on the causation of the development on this tumor, but the role of each of them on the initiation of the tumor is still debated. The resolution of this question could have important consequences, both in term of recommendations of sanitary authorities and development of effective sunscreens. The discussion is so controversial that few studies even brought the benefits of sunscreens into question and explained that, due to a nonprotection against UVA, they might be ineffective [30, 31] and, as a indirect consequence, contribute to increase melanoma incidence [32, 33]. Some other epidemiological studies suggested that both UVA and UVB were involved in melanoma causation [34–37], whereas nonmelanoma skin cancers have primarily linked to UVB [38]. These results suggest that melanin could play a role in the melanoma sensitization to UVA. 

This role of melanin was put in evidence when Sarna and Sealy demonstrated that the exposition of melanin to UV and blue light generates reactive melanin radicals (RMRs). Moreover, it was demonstrated that RMR could react with biomolecules and molecular oxygen [39, 40] to lead to the formation of oxygen reactive species, such as superoxide, leading to hydrogen peroxide and hydroxyl radical. 

An EPR technique was recently developed by Wood et al. [41] to enable accurate measurement of RMR *in situ* in skin. This method was used to assess quantitatively the RMR formation in function of the exposition to different wavelength in the skin of a mouse model for which the action spectrum was already known [30]. They observed that the 2 action spectra were identical from 303 to 434 nm, a range spanning both UVB and UVA. This result demonstrated that the EPR measurement of reactive melanin radicals could act as good indicator to determine the contribution of UVA and UVB in melanoma causation. In this study, it was shown that over 95% of measured RMR were caused by UVA, with less than 5% by UVB. Consequently, a sunscreen that would block the major part of UVB, but would be ineffective versus UVA, would offer very limited protection against RMR formation.

These results still have to be confirmed on other animal species (including human), and we can predict that the controversy about UVA, UVB, and melanoma is far away from the end, but these preliminary results could bring the outcome faster than expected.

## 5. The Detection and Growth State Assessment of Melanoma by EPR

The first important work concerning the quantitative detection of melanin by EPR spectrometry inside melanoma samples was published by Elek et al. in 1980. When measuring ocular melanomas embedded in paraffin, they observed a free-radical signal situated at g-factor = 2.003, corresponding to the EPR signal of melanin [42]. When comparing the amplitude of this signal with the number of melanin granules observed in histological sections, they observed a straight positive correlation. They concluded from this experiment that EPR spectrometry might be suitable for estimating the melanin content inside melanoma samples.

This study was followed in 1990 by Katsuda et al. who adapted a self-made EPR cavity to an EPR imaging device and managed to get the first EPR image of endogenous pigments inside *ex vivo* melanoma [43]. However, an EPR image of melanoma had been obtained *in vivo* three years before by injecting a paramagnetic nitroxide contrast agent [44] near the tumor. This image was actually the first EPR image of melanoma but was not the reproduction of the repartition of melanin, but of the nitroxide contrast agent.

In 2005, after many years of work using EPR to study the properties of melanin and the effects of UV's on melanin and melanoma development, Timmins had the premention that EPR imaging could be helpful in the melanoma diagnosis and submitted a patent for the general concept of “detecting melanin by EPR” [45].

**Figure 3:**
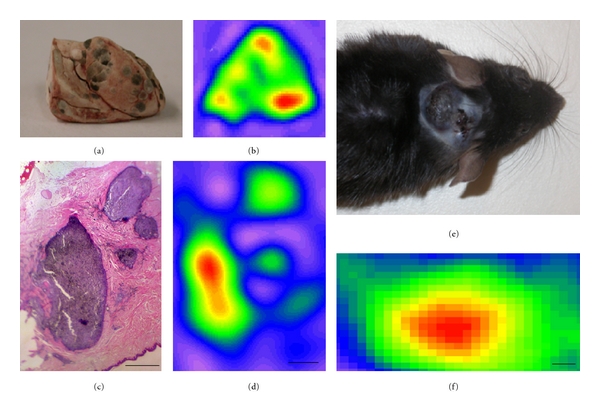
(a, b) Melanoma B16 metastases in the lungs of mice: picture of freeze-dried lungs with metastases (a) and the respective 2D transversal EPR image (b). (c, d) 2D EPR image through a section of thickness 500 mm of a paraffin-embedded human melanoma (d) and the histological section (5 mm thick) from a contiguous slice (c). Scale bars: 1 mm. (e, f) *In vivo* studies on B16 melanoma in mice. Melanoma grown in subcutaneous tissue (e) and *in vivo* EPR image obtained using a low-frequency EPR spectrometer with a head-coil loop-gap resonator. Scale bar: 2 mm. Pictures coming from Vanea et al. [15].

In 2008, Vanea et al. [15] assessed the potential of EPR to image freeze-dried mouse cutaneous melanomas and lung metastases. The freeze-drying process was required to avoid a nonresonant absorption of the microwaves by the water in the 9 GHz EPR system. They observed that the 2D and 3D EPR images were fitting very well with the visual aspect of the melanoma samples and invaded lungs (Figures 3(a), and 3(b)).

In the same study, they adapted their method to the measurement of paraffin-embedded human melanomas. As a result, they were able to correlate EPR images of these human melanomas with contiguous histological sections coming from the same tumors (Figures 3(c), and 3(d)) and demonstrate fair accuracy of EPR imaging.

In the final part of their work, they were able to obtain an EPR image *in vivo* of a large mouse melanoma B16 model, using a low microwave frequency EPR imager. This image was the first EPR image of the endogenous melanin pigments inside of an *in vivo* melanoma (Figures 3(e), and 3(f)).

Consecutively to the work of Vanea et al., it was decided to investigate the relationship between the growth state of mouse B16 cutaneous melanomas or metastases expressing luciferase, measured by the bioluminescence imaging (BLI) technique and their EPR spectra [15, 46]. In this study, we showed that there was a straight correlation between the EPR intensity of the signal, reflecting the melanin content of the tumor or invaded lung, and the bioluminescence intensity corresponding to the same sample. The same study was made on KHT fibrosarcomas (nonpigmented) so that the predominant role of the presence of melanin on the spectrum was confirmed. 

By comparing the two methods, it was moreover demonstrated that EPR spectrometry was more accurate than BLI in terms of assessment of the tumoral growth level. These results, even if very encouraging, might however be moderated as BLI measurements were performed *in vivo*, while EPR measurements were performed *ex vivo.*

Finally, the limit of detection of the method in the present configuration (EPR Bruker E540 Elexsys equipped with a super high-sensitivity probe for X-Band (9 GHz) measurements and equipped with an E540R23 L-B and EPR head-coil resonator for L-Band (1.1 GHz) was assessed. This experiment, made in parallel with synthetic melanin and melanoma powder, allowed us to determine that such a tiny quantity like 2 *μ*g of melanin could be detected *ex vivo* in favorable conditions using X-Band EPR spectrometry (system dedicated to *in vitro* or *ex vivo* studies; large freeze-dried samples, or aqueous samples with a thickness of les than 1 mm). The detection threshold was however around 10^3^ times higher for L-Band and measurements (adapted for *in vivo* studies; 1 cm depth penetration). Consequently, the performances of the method should still be improved to allow accurate and sensitive measurements *in vivo*.

## 6. Discussion and Conclusion

For approximately fifty years, considerable progresses were performed in the field of melanin and melanoma knowledge. These progresses are notably due to advances in biochemistry, genetics, and molecular biology. Parallel to these advances, technological developments in the field of magnetic resonance were achieved so that, nowadays, it is possible to apply the electron paramagnetic resonance spectroscopy and imaging to the detection of biological-free radicals, including those trapped in melanins, with a high sensitivity.

The application of EPR to melanins has led to many discoveries concerning melanin structure and melanoma development. As shown by Lund and Timmins [6], EPR spectrometry appeared as a unique tool to identify the ultraviolet range responsible of melanoma causation, and, by the way, to help to resolve a thirty-year-old controversy. We can expect that the consequences of this demonstration will have important consequences in the field of sunscreen development, which should undoubtedly contribute to a reduction of melanoma prevalence.

On the other hand, the improvements achieved in the field of EPR spectrometry during these last years allowed us to measure accurately and sensitively the presence of melanin pigments inside melanoma samples. As a straight correlation was found between the intensity of the EPR signal of melanin and the tumor growth state, the signal of melanin appears as a good indicator for melanoma development. Moreover, due to a continuous improvement of EPR imager performances, the first *in vivo* mapping of endogenous melanin pigments inside melanoma could recently be achieved by EPR imaging. Ongoing researches are now focusing on the characterization of human melanoma samples by this technique. Indeed, the diagnosis of melanoma in human by the ABCDE optic rule, even if effective, does not refer to any objective quantifiable standard. Moreover, an important limitation of the technique is the impossibility to obtain information about the tumor penetration (Clark's index) without performing a biopsy. The use of EPR imaging could fill in these lacks, that is why this method is at present tested on *ex vivo* human melanoma samples with different Clark's index. Applied *in vivo*, it would help the surgeon to define more precisely the margins for lesions resections, based on a noninvasive method. Preliminary results are encouraging, but it seems that early melanomas could provide an EPR signal that is insufficient for the reconstruction of EPR images. If these results are confirmed, new technical improvements in terms of magnetic gradient field or EPR cavity should be required before adapting the method to *in vivo* measurements.

## References

[1] Longstreth J (1988). Cutaneous malignant melanoma and ultraviolet radiation: a review. *Cancer and Metastasis Review*.

[2] Woodhead AD, Setlow RB, Tanaka M (1999). Environmental factors in nonmelanoma and melanoma skin cancer. *Journal of Epidemiology*.

[3] Armstrong BK (1988). Epidemiology of malignant melanoma: intermittent or total accumulated exposure to the sun?. *Journal of Dermatologic Surgery & Oncology*.

[4] Gandini S, Sera F, Cattaruzza MS (2005). Meta-analysis of risk factors for cutaneous melanoma: II. Sun exposure. *European Journal of Cancer*.

[5] Whiteman DC, Stickley M, Watt P, Hughes MC, Davis MB, Green AC (2006). Anatomic site, sun exposure, and risk of cutaneous melanoma. *Journal of Clinical Oncology*.

[6] Lund LP, Timmins GS (2007). Melanoma, long wavelength ultraviolet and sunscreens: controversies and potential resolutions. *Pharmacology and Therapeutics*.

[7] Berzelius JJ (1840). *Lehrbuch der Chemie*.

[8] Riley PA (1997). Molecules in focus: melanin. *The International Journal of Biochemistry & Cell Biology*.

[9] Nicolaus RA (1968). *Melanins*.

[10] Prota G, Nicolaus RA (1967). *Advances in Biology of Skin*.

[11] Prota G, Rorsman H, Rosengren AM, Rosengren E (1976). Phaeomelanic pigments from a human melanoma. *Experientia*.

[12] Rorsman H, Agrup G, Hansson C, Rosengren AM, Rosengren E, Klaus SN (1979). Detection of pheomelanin. *Pigment Cell*.

[13] Gerlach W, Stern O (1922). Das magnetische moment des silberatoms. *Zeitschrift für Physik*.

[14] Uhlenbeck GE, Goudsmit S (1926). Spinning electrons and the structure of spectra. *Nature*.

[15] Vanea E, Charlier N, DeWever J (2008). Molecular paramagnetic resonance imaging of melanin in melanomas: a proof-of concept. *NMR in Biomedicine*.

[16] Gallez B, Baudelet C, Jordan BF (2004). Assessment of tumor oxygenation by electron paramagnetic resonance: principles and applications. *NMR in Biomedicine*.

[17] Cammack R, Cooper CE (1993). Electron paramagnetic resonance spectroscopy of iron complexes and iron- containing proteins. *Methods in Enzymology*.

[18] Vikram DS, Zweier JL, Kuppusamy P (2007). Methods for noninvasive imaging of tissue hypoxia. *Antioxidants and Redox Signaling*.

[19] Kleschyov AL, Wenzel P, Munzel T (2007). Electron paramagnetic resonance (EPR) spin trapping of biological nitric oxide. *Journal of Chromatography B*.

[20] Villamena FA, Zweier JL (2004). Detection of reactive oxygen and nitrogen species EPR spin trapping. *Antioxidants and Redox Signaling*.

[21] Swartz HM, Khan N, Khramtsov VV (2007). Use of electron paramagnetic resonance spectroscopy to evaluate the redox state in vivo. *Antioxidants and Redox Signaling*.

[22] Hoch MJR, Day AR (1979). Imaging of paramagnetic centres in diamond. *Solid State Communications*.

[23] Karthe EW, Wehrsdorfer E (1979). The measurement of inhomogeneous distributions of paramagnetic centers by means of EPR. *Journal of Magnetic Resonance*.

[24] Fujii H, Sato-Akaba H, Kawanishi K, Hirata H (2011). Mapping of redox status in a brain-disease mouse model by three-dimensional EPR imaging. *Magnetic Resonance in Medicine*.

[25] Kuppusamy P, Shankar RA, Zweier JL (1998). In vivo measurement of arterial and venous oxygenation in the rat using 3D spectral-spatial electron paramagnetic resonance imaging. *Physics in Medicine and Biology*.

[26] Jordan BF, Misson P, Demeure R, Baudelet C, Beghein N, Gallez B (2000). Changes in tumor oxygenation/perfusion induced by the no donor, isosorbide dinitrate, in comparison with carbogen: monitoring by EPR and MRI. *International Journal of Radiation Oncology Biology Physics*.

[27] Bratasz A, Pandian RP, Deng Y (2007). In vivo imaging of changes in tumor oxygenation during growth and after treatment. *Magnetic Resonance in Medicine*.

[28] Levêque P, Godechal Q, Bol A, Trompier F, Gallez B (2009). X-band EPR imaging as a tool for gradient dose reconstruction in irradiated bones. *Medical Physics*.

[29] Commoner B, Townsend J, Pake GE (1954). Free radicals in biological materials. *Nature*.

[30] Setlow RB, Grist E, Thompson K, Woodhead AD (1993). Wavelengths effective in induction of malignant melanoma. *Proceedings of the National Academy of Sciences of the United States of America*.

[31] Setlow RB (1999). Spectral regions contributing to melanoma: a personal view. *Journal of Investigative Dermatology Symposium Proceedings*.

[32] Garland CF, Garland FC, Gorham ED (1993). Rising trends in melanoma. An hypothesis concerning sunscreen effectiveness. *Annals of Epidemiology*.

[33] Setlow RB, Woodhead AD (1994). Temporal changes in the incidence of malignant melanoma: explanation from action spectra. *Mutation Research*.

[34] Moan J, Dahlback A, Setlow RB (1999). Epidemiological support for an hypothesis for melanoma induction indicating a role for UVA radiation. *Photochemistry and Photobiology*.

[35] Oliveria S, Dusza S, Berwick M (2001). Issues in the epidemiology of melanoma. *Expert Review of Anticancer Therapy*.

[36] Wang SQ, Setlow R, Berwick M (2001). Ultraviolet A and melanoma: a review. *Journal of the American Academy of Dermatology*.

[37] Garland CF, Garland FC, Gorham ED (2003). Epidemiologic evidence for different roles of ultraviolet A and B radiation in melanoma mortality rates. *Annals of Epidemiology*.

[38] de Gruijl FR, Forbes PD (1995). UV-induced skin cancer in a hairless mouse model. *BioEssays*.

[39] Sarna T, Sealy RC (1984). Free radicals from eumelanins: quantum yields and wavelength dependence. *Archives of Biochemistry and Biophysics*.

[40] Sarna T, Sealy RC (1984). Photoinduced oxygen consumption in melanin systems. Action spectra and quantum yields for eumelanin and synthetic melanin. *Photochemistry and Photobiology*.

[41] Wood SR, Berwick M, Ley RD, Walter RB, Setlow RB, Timmins GS (2006). UV causation of melanoma in Xiphophorus is dominated by melanin photosentisized oxidant production. *Proceedings of the National Academy of Sciences of the United States of America*.

[42] Vivo-Acrivos JF, Blois MS An electron spin resonance study of stable free radicals in natural and synthetic melanins.

[43] Katsuda H, Kobayashi T, Saito H, Matsunaga T, Ikeya M (1990). Electron spin resonance imaging of mouse B16 melanoma. *Chemical and Pharmaceutical Bulletin*.

[44] Berliner LJ, Fujii H, Wan XM, Lukiewicz SJ (1987). Feasibility study of imaging a living murine tumor by electron paramagnetic resonance. *Magnetic Resonance in Medicine*.

[45] Timmins GS

[46] Godechal Q, Defresne F, Danhier P (2011). Assessment of melanoma extent and melanoma metastases invasion using electron paramagnetic resonance and bioluminescence imaging. *Contrast Media and Molecular Imaging*.

